# Reap success from persistence

**DOI:** 10.7189/jogh.12.03077

**Published:** 2022-12-21

**Authors:** Cuihong Tian, Jingyi Yan, Xuerui Tan, Wei Wang

**Affiliations:** 1 Clinical Research Centre, The First Affiliated Hospital of Shantou University Medical College, Shantou, Guangdong, China; 2 Centre for Precision Health, Edith Cowan University, Perth, Australia; 3 Department of Cardiovascular Medicine, The First Affiliated Hospital of Shantou University Medical College, Shantou, Guangdong, China

The road to success is long and arduous. Almost all Nobel prize laureates experienced tremendous efforts and countless failures before they made their scientific breakthroughs. Hypothesis-driven, independent and critical thinking, passion, repeated experiments and repetitive failures and running in circles on the entire scientific process finally approved their hypotheses.

## DRIVEN BY INTEREST, FOCUSING ON DIRECTION

As a milestone, Charles Louis Alphonse Laveran (French) fired the first shot using science against malaria, after mankind had been suffering from this illness for thousands of years [1]. Previously, many investigators failed to elucidate the cause of malaria as they examined the air, water, or soil in malaria-endemic areas. However, Laveran paid more attention to the pathological anatomy and blood tests of malaria patients. He began his research on malarial parasites in Bône, Algeria, where innumerable deaths from malaria provided him with an opportunity to study malaria under natural conditions. Highly intrigued by this ferocious, intermittent high-fever and chill disease, he began to dissect patients who died of malaria to hunt for its aetiology. Using (at the time) poor blood examination technology, Laveran found that the blood of those deceased from malaria was filled with large amounts of black pigment resembling flagellated microorganism that moved rapidly, either freely or adherent to red blood cells. Finally, he verified that this microorganism causing melanemia is a parasite, and named it malaria. He subsequently delineated its process of growth, proliferation, and invasion in vivo. Henceforth, people gave up blaming putrefying air for malaria thanks to Laverne’s discovery.

## HEADING DEEP INTO THE EPIDEMIC AREA, DANCING WITH MOSQUITOES

Fearless persistence also contributed to the success of Ronald Ross (British) [2]. Although Ross won the Nobel prize before Laveran, his research was launched after the discovery of the malaria pathogen. Many researchers had suspected that mosquitoes were the intermediate hosts for malaria transmission, but they failed to prove the hypothesis with convincing evidence. Driven by a medical practitioner’s mission and responsibility, Ross headed deep into malaria epidemic areas and caught mosquitoes by himself for his experiments. Due to poor protective conditions, Ross suffered from cholera and malaria successively, almost losing his life. Stimulated by his original intentions, Ross studied dozens of hypotheses by dissecting hundreds of species of mosquitoes and examining every mosquito tissue, but found no compelling proof. Ross realized that dissecting mosquitoes in water or a weak salt solution left him less time for methodical staining. He then tried to dissect the mosquitoes in a concentrated salt solution and discovered the presence of spores. Through extensive observation, Ross found that the spores in the mosquito’s body were attached to a duct and encapsulated to form a part of the salivary glands, which injected saliva into the blood when the mosquito bites the skin. The discovery of malaria vectors shattered the entrenched miasma theory. Malaria, which once rendered humans helpless, finally turned out to be a preventable and controllable disease.

**Figure:**
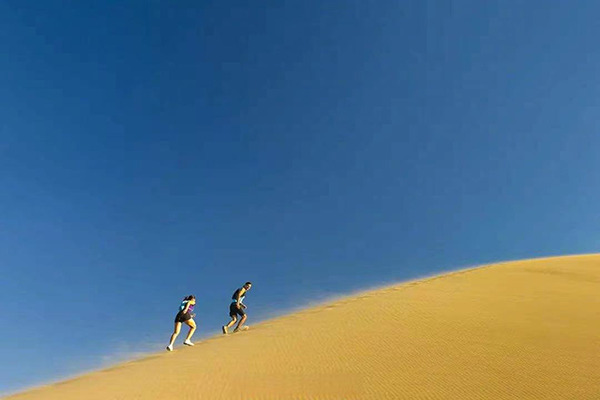
Photo: Success lies in persistence. Source: Copyright free, available at: https://mbd.baidu.com/newspage/data/dtlandingwise?nid=dt_4268703082288917219&sourceFrom=homepage.

## INSPIRED BY CASES, INNOVATING BOLDLY

Unexpectedly, malaria, which was once a source of evil, was found to have a beneficial effect for mankind via the long-term insistent research. An epidemic of syphilis occurred in 1700s and 1800s, resulting in syphilitic patients dying of paralytic dementia, an advanced symptom of syphilis. However, there were cases of advanced patients whose symptoms disappeared and consciousness returned to normal after suffering from a febrile infectious disease, which inspired Wagner-Jauregg (Austrian) who came up with a bold idea of treating paralytic dementia by inducing fevers [3]. Through excessive trials and long-term follow-up, he eventually showed that inoculating malaria enables relief from the symptoms of paralytic dementia. Jauregg speculated that the recyclable and favorable repair process caused by malaria in patients with paralytic dementia was the main principle of malaria vaccination therapy to treat paralytic dementia. Although phased out with the advent of penicillin, malaria inoculation was widely used for the treatment of paralytic dementia and saved thousands of lives during the 1960s.

## EXPLORING THE UNKNOWN, TO KEEP ON FIGHTING

Paul Hermann Müller (Swiss) experienced a total of 349 failures while exploring synthetic pesticides [4]. The early 1900s witnessed the outbreaks of a food crisis, typhus, and malaria. The expense of natural insecticides, such as pyrethroids and rotenone, limited their widespread use in agriculture, resulting in an urgent need for new synthetic insecticides. Müller struggled in his research on agricultural pesticides, as nearly everything in this area was unknown at the time. He tested hundreds of different substances, but all the results were unsatisfactory. However, Müller’s experiments were not all valueless. For example, he found that insecticides containing a -CCl3 chemical group had considerable contact insecticidal activity against flies. He then prepared derivatives based on this basic chemical group, and eventually synthesized dichlorodiphenyltrichloroethane (DDT), which was widely applied in agriculture due to simple production, powerful toxicity, and low price. Although DDT was later banned due to its negative impact on the environment and food chain, we cannot deny its prominent role both in malaria and agricultural pest control during World War II (1939-1945).

## FATE TIES TU TO ARTEMISININ

Even distant dreams can come true through insistent perseverance. Previously, chloroquine had been an effective medicine for malaria treatment. However, falciparum malaria, a type of malaria plasmodium parasite, was resistant to chloroquine. Youyou Tu, a Chinese pharmacologist, started a comprehensive search for antimalarial drugs in 1969 [5]. She consulted various traditional Chinese medicine classics and folk remedies, interviewed many experienced Chinese medicine practitioners, and extracted over a hundred different herbs for animal experiments, but all failed. Subsequently, Tu re-reviewed the classics of Chinese medical literature, and one sentence, “A handful of artemisinin immersed in two liters of water, wring out the juice and drink it all” made Tu realize that temperature may have a significant impact on the herbs’ medicinal activity. Therefore, Tu re-extracted artemisinin at a lower temperature and found that the artemisinin ethyl ether extract achieved 100% effectiveness in the killing malaria. This discovery was of great value, not only on saving millions of malaria patients’ lives, but also serving as an evidence-based example of Chinese herbs for disease treatment.

## DISCUSSION

**Figure 1:**
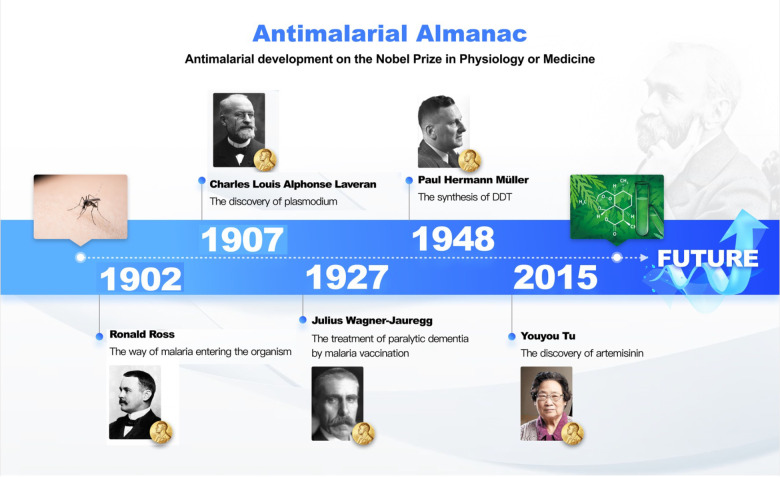
Five scientists were awarded the Nobel prize in physiology or medicine. DDT – dichlorodiphenyltrichloroethane.

The relay of several generations of Nobel prize winners’ work on malaria verifies that persistence is key to success (**Figure 1**). Their rigorous scientific attitudes and persistent stamina for scientific research are an example for the new generation of researchers. Each of these five scientists’ Nobel-prize winning discoveries was closely related to the long-term malaria epidemic’s historical background, their enduring struggles, and their persistent, decades-long research to approve their hypotheses. Every major discovery or breakthrough is inseparable from interest, curiosity, practice, innovation, adventure, and persistence. This battle against malaria shows that the road towards success has been long and obstructed. However, persistence will always be the key ingredient for success.

Exploring new antimalarial approaches remains imperative due to continuous emergence of malarial resistance. Scientists who successfully develop an effective malarial vaccine or apply genome editing to sterilize mosquitoes would cause a sensation worldwide and be awarded further Nobel prizes. Since the first malaria vaccine reported in 1973 [6], many candidate vaccines have undergone clinical assessment [7]. For example, one of the malaria vaccine candidates, R21 in adjuvant Matrix-M (R21/MM), applied in Phase II clinical trials in Africa, has been demonstrated to be safe and immunogenic with promising high-level efficacy [8]. Genetic editing, CRISPR-Cas9, targeting female sterilization in Anopheles gambiae (the main mosquito vector for malaria), shows great potential in controlling malaria transmission by suppressing mosquitoes [9]. The combination of malaria vaccination and genetic editing is expected to considerably reduce the incidence and case fatality rate of malaria. Additionally, people with heterozygous sickle cell disease were observed to seldom suffer from malaria due to the weak oxygen-carrying capacity of red blood cells, which contributes to a 90% risk drop of malaria in African children [10], offering promising prospects to malaria control. Growing knowledge, progressive methods, and tireless efforts of several generations suggest malaria will be ended in the near future.

The entire antimalarial journey verifies that no shortcuts exist in scientific development. Many difficulties in developing malaria vaccine, such as the technical complexity of vaccination against parasite, the lack of effective animal models, and the coinfection of multiple plasmodia, remain to be overcome. Genetic editing also raises challenges on ethics and natural selection theory. It is still believed that those scientists concentrating on the correct research direction (ie, malaria vaccine or mosquitoes’ sterilization to fight against malaria) will reap success from their great efforts and persistence.
